# A Scoping Review with Bibliometric Analysis of Para-Rowing: State of the Art and Future Directions

**DOI:** 10.3390/healthcare11060849

**Published:** 2023-03-13

**Authors:** Luca Puce, Carlo Biz, Carlo Trompetto, Lucio Marinelli, Antonio Currà, Luca Cavaggioni, Matteo Formica, Vittorio Vecchi, Maria Chiara Cerchiaro, Khaled Trabelsi, Nicola Luigi Bragazzi, Pietro Ruggieri

**Affiliations:** 1 Department of Neuroscience, Rehabilitation, Ophthalmology, Genetics, Maternal and Child Health (DINOGMI), University of Genoa, 16132 Genoa, Italy; 2 Orthopedics and Orthopedic Oncology, Department of Surgery, Oncology and Gastroenterology (DiSCOG), University of Padova, 35128 Padova, Italy; 3 IRCCS Ospedale Policlinico San Martino, 16132 Genoa, Italy; 4 Academic Neurology Unit, A. Fiorini Hospital, 04019 Terracina, Italy; 5 Department of Biomedical Sciences for Health, Università Degli Studi di Milano, 20129 Milan, Italy; 6 Obesity Unit and Laboratory of Nutrition and Obesity Research, Department of Endocrine and Metabolic Diseases, Istituto Auxologico Italiano IRCCS, 20145 Milan, Italy; 7 Orthopedic Clinic, Department of Integrated Surgical and Diagnostic Sciences (DISC), University of Genoa, 16132 Genoa, Italy; 8 Research Laboratory: Education, Motricity, Sport and Health, EM2S, LR19JS01, University of Sfax, Sfax 3000, Tunisia; 9 High Institute of Sport and Physical Education, University of Sfax, Sfax 3000, Tunisia; 10 Laboratory for Industrial and Applied Mathematics (LIAM), Department of Mathematics and Statistics, York University, Toronto, ON M3J 1P3, Canada

**Keywords:** paralympic sport, para-rowing, scoping review, bibliometric analysis

## Abstract

Para-rowing is a format of rowing practiced by people with different types of disabilities, thanks to adapted equipment set-ups and regulations. Para-rowing made its debut recently at the 2008 Paralympic Games. According to the mandate of the “International Paralympic Committee”, para-rowers should be enabled to pursue sporting excellence. Therefore, rigorous research is needed in terms of well-designed, high-quality studies. To the best of our knowledge, there are no systematic appraisals of the body of scholarly evidence in the field of para-rowing. As such, a scoping review enhanced by bibliometric analyses was carried out to provide a comprehensive synthesis of knowledge related to para-rowing for the perusal of practitioners and athletes. By mining eighteen major databases, 17 studies were retained in the present review. The included studies were found to focus on a range of aspects involving health, the etiology of injuries (n = 5), psychological and physiological responses (n = 5), performance, biomechanical analysis (n = 4), and new analytical approaches for kinematic assessments and predictions of mechanical outputs in para-rowers (n = 3). The scholarly community on para-rowing consists of 78 researchers, 16 (20.51%) of whom are highly interconnected. The most prolific author was Smoljanović T., from Croatia, with three items/documents. In total, 93.6% of scholars have authored one single document. Topological features indicated a highly fragmented and dispersed, poorly connected community characterized by a high number of clusters and a low strength of connections. In terms of publication years, the first scholarly article dates back to 2008, with four articles (23.5%) published in the current year, showing an increasing interest in this para-sports discipline. Finally, gaps in current research on para-rowing were identified in terms of overlooked topics, including sports nutrition, doping, and psychological aspects in para-rowers other than those with visual impairment.

## 1. Introduction

Para-rowing or adaptive rowing or adaptive sculling is a format of rowing practiced by people with different types of disabilities, especially thanks to adapted equipment set-ups and regulations [1]. These adaptations and/or technical modifications are needed to facilitate its practice in the presence of impairments in muscle and joint functions (i.e., strength, or range of motion, ROM), movement deficiencies (athetosis/hypertonia/ataxia), differences in physical structure (lower limb deficiency/amputation), and/or a range of sensory and physical disabilities (visual impairment, orthopedic impairments, and upper/lower motor neuron syndrome) [2]. Para-rowing made its debut for the first time, at the 2008 Paralympic regattas in Beijing, in 1000 m races. This distance was adopted at the 2012 and 2016 Paralympic games, when, in 2017, a new rule change doubled the distance. At the Tokyo 2020 Paralympic games, para-rowing used the same distance as its Olympic counterpart—2000 m. According to the para-rowing classification regulations of the World Rowing Association [3], para-rowers are assigned to three different sport classes based on sex/gender and level of physical function: PR1 (with minimal or no trunk function, with preserved arm and shoulder function to propel the boat, and with poor sitting balance, for which they require to be strapped to the boat/seat), PR2 (with functional use of arms and trunk, but weak/absent leg function to slide the seat), or PR3 (with residual function in the legs which allows para-rowers to slide the seat). PR3 functional class also includes rowers with intellectual and vision impairment. Para-rowing can be practiced either individually or as a team discipline, composed of two or four para-rowers. Specifically, there are four events: two singles (PR1 Men’s Single Sculls—PR1M1x and PR1 Women’s Single Sculls—PR1W1x), one of a man and woman couple (PR2 Mixed Double Sculls—PR2Mix2x) and one with a double couple of two men and two women (PR2 Mixed Double Sculls—PR2Mix2x). The hulls of para-rowing boats are generally larger and heavier than able-bodied single and double sculls and are equipped with special seats that vary according to the athlete’s disability. For example, in the PR1 and PR2 sports classes, the boats have fixed seats with backrests. Sometimes, in the case of athletes belonging to the PR1 sports class, the boats can have further modifications and be equipped with flotation systems that act as stabilizers (known as pontoons) and are fixed to the oarlocks of the boat to ensure further lateral balancing (optional for PR2 rowers). Further adaptions can be represented by strapping: chest straps are needed for PR1 rowers for increased stability, whereas knee straps are used for PR2 rowers to prevent any flexion or extension of the knee during rowing. Able-bodied rowers apply techniques that maximize the drive of the legs, followed by the back and arms in a synchronized manner [4]. When parts of this kinetic chain are lost due to physical impairments, these techniques cannot be implemented. This results in decreased performance and impaired rowing technique by the para-rowers especially in sports classes PR1 and PR2 [5]. For example, the PR1 rowers’ current world records are approximately 3 min slower (about 7 vs. 10 min) than their counterparts without physical disabilities [6]. Furthermore, the former has a higher stroke rate at the expense of a shorter stroke length [7]. However, competition times and time discrepancies between sports classes have improved a lot in recent years probably due to the growing technical and scientific support for these athletes [8]. Finally, despite the disability-induced fatigue, para-athletes undergo training frequencies, volumes, and intensities very similar to able-bodied athletes of the same level [9].

According to the mandate of the “International Paralympic Committee” (IPC), para-rowers should be enabled to pursue sporting excellence [10]. Therefore, rigorous research is needed in terms of well-designed studies. To the best of our knowledge, there are no systematic appraisals of the body of scholarly evidence in the field of para-rowing. The existing body of literature includes anecdotal stories and personal experiences, as well as single case reports and manuals/handbooks. There exist reviews on rowing, but, in para-rowing, the areas of the athlete’s body that are subjected to higher force transmission are altered [5]. 

As such, given the unique challenges of para-rowing, this investigation was carried out to provide a comprehensive synthesis of knowledge related to this para-sports discipline for the perusal of both practitioners and athletes, to better inform training protocols and conditioning strategies, as well as for the para-rowing scholarly community, to offer guidance for future studies and research.

## 2. Materials and Methods

### 2.1. Study Protocol, Study Conceptual Design, and Development

An a priori study protocol was drafted before formally commencing the study research, but it could not be submitted and registered within the “International prospective register of systematic reviews” (PROSPERO,) in that PROSPERO does not accept anymore scoping reviews or literature scans. Given our research aims, the present literature review was performed and designed as a scoping review enhanced by bibliometric analyses [11]. Arksey and O’Malley’s five-step methodology [12] was followed as well as the “Patterns-Advances-Gaps-Evidence for Practice-Recommendations” (PAGER) framework developed by Bradbury-Jones et al. [13]. More in detail, the aim was to synthesize the body of currently available research on para-rowing in terms of evidence, knowledge and practice gaps, recommendations and policies, prospects, and directions in the field, for the benefit of both the sports and scholarly communities.

Arksey and O’Malley’s five-step methodology [12] consists of (i) identifying and developing the research question(s), (ii) identifying the body of relevant scholarly studies, (iii) selecting the studies to retain in the review, based on well-defined inclusion/exclusion criteria, (iv) extracting and charting the data (either quantitative or qualitative), and (v) collating, summarizing, and reporting the major findings of the studies included in the present review.

Building on the previous steps, the PAGER framework [12] consists of (i) synthesizing the key findings in terms of unique key themes/thematic areas (P), (ii) discovering and dissecting the dynamic underlying these themes (A), (iii) identifying under-developed/overlooked themes that warrant future research (G), (iv) advising relevant stakeholders from the sports and scholarly communities and informing/shaping practices (E), and (v) guiding future research (R).

### 2.2. Identification of Relevant Studies

The following keywords were used: “adaptive rowing”, “adaptive sculling”, “para rowing”, “para rower”, “para rowers”, “paralympic rower”, and “paralympic rowers”. These keywords were combined in a search string using the “OR” Boolean connector. Eighteen major electronic, scholarly databases (namely, Scopus, Gale Academic OneFile, IngentaConnect Journals, ProQuest Central, Science Citation Index Expanded (Web of Science), PubMed Central, Factiva, ROAD or Directory of Open Access Scholarly Resources, Journals@Ovid Complete, Taylor and Francis Journals Complete, Taylor and Francis CRKN Social Science and Humanities, Web of Science, Taylor & Francis CRKN Science and Technology, Nursing and Allied Health Database, Web of Science—Science Citation Index Expanded—2022, Scholars Portal Journals: Open Access, Web of Science—Social Sciences Citation Index—2022, and Scholars Portal Journals) were mined from inception using the Omni academic search tool, without language filters/restrictions. The search was conducted up to 17 February 2022. Citation information was exported using the “Research Information Systems” (RIS) file format, which is a standardized tag format developed to enable citation programs to exchange and share data related to citation information.

### 2.3. Study Selection and Inclusion/Exclusion Criteria

As recommended by the Joanna Briggs Institute (JBI), the Population (or Participants), Concept, and Context (PCC) mnemonic was employed as a guide to constructing a clear and meaningful title and framing and developing the subsequent research question(s). 

Inclusion and exclusion criteria [14] were the following: studies focusing on para-rowing and with athletes, exposed to any kind of interventional strategy (if any, either a warm-up or training/conditioning program, nutritional supplementation, pharmacological intervention, recovery strategy, etc.), compared against other disabled or able-bodied athletes, assessed in terms of age, sex/gender, functional sport class, years of experience and training, competing level—regional, national, international-, type of disability/impairment and if congenital/acquired). Studies were retained if they focused on any outcome relevant to para-rowing (kinematic, biomechanical, physiological, psychological or psycho-physiological, epidemiological, methodological, etc.). Any study design was deemed eligible: any original study with sufficient (quantitative/qualitative) details was scrutinized. Reviews were not included but were scanned to increase the chance of finding any relevant study, whereas commentaries, letters to the editor, editorials, expert opinions, or technical notes without sufficient details were discarded. Articles were also excluded if focusing on other Paralympic disciplines or reporting data in such a way that it was not possible to disaggregate them, and extract data related to para-rowing only. Finally, non-peer-reviewed items (including conference proceedings and abstracts, theses/dissertations, (e)books/(e)book chapters, newsletter articles, and reports, among others) were not deemed eligible. 

### 2.4. Bibliometrics Analysis

Using ad hoc extraction, processing, visualization, and bibliometric software, including VOSviewer version 1.6.18, Gephi, and Cytoscape [15], data extracted from MEDLINE via PubMed and RIS reference manager files were mapped and visualized as graphs/networks and investigated from a quantitative standpoint, by computing a range of several graph theory/network-related indicators. Finally, the number of papers per year was also visualized as a time series. Further details are described in our previous publication, to which the reader is referred [15].

## 3. Results

### 3.1. Literature Search

The initial literature search yielded a pool of 157 items (n = 18 from Scopus, n = 17 from Gale Academic OneFile, n = 15 from IngentaConnect Journals, n = 14 from ProQuest Central, n = 12 from Science Citation Index Expanded (Web of Science), n = 11 from PubMed Central, n = 8 from Factiva, n = 8 from ROAD, n = 7 from Journals@Ovid Complete, n = 7 from Taylor & Francis Journals Complete, n = 6 from Taylor & Francis CRKN Social Science and Humanities, n = 6 from Web of Science, n = 6 from Taylor & Francis CRKN Science and Technology, n = 5 from the Nursing and Allied Health Database, n = 5 from Web of Science—Science Citation Index Expanded—2022, n = 4 from Scholars Portal Journals: Open Access, n = 4 Web of Science—Social Sciences Citation Index—2022, and n = 4 from Scholars Portal Journals).

**Figure 1:**
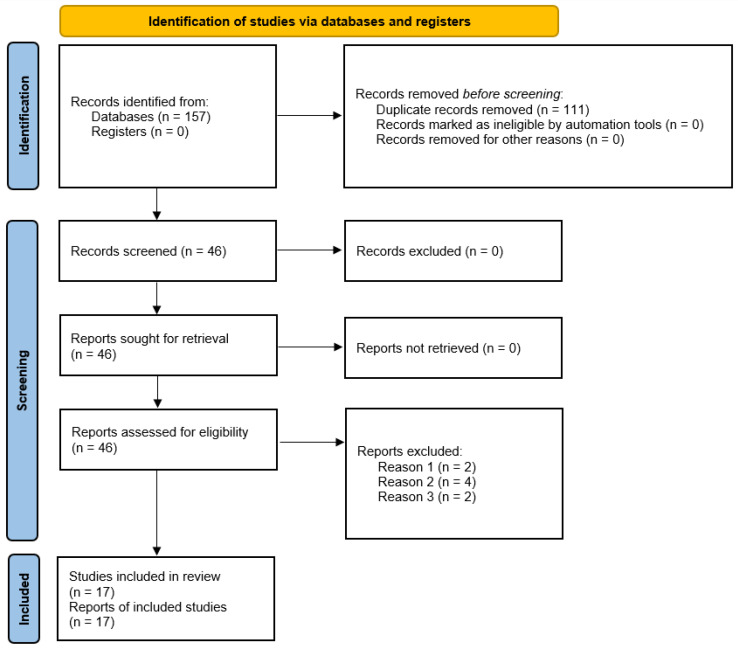
Flowchart adopted in the present scoping review.

A total of 111 items were duplicated and were, as such, removed, and 46 items were inspected, by looking at the title and/or abstract. Based on the inclusion/exclusion criteria, eleven items were discarded based on their study design/format (newsletter articles (n = 4), books and ebooks (n = 3), theses and dissertations (n = 2), reports (n = 1), book chapters (n = 1)). Out of 35 studies, further ten studies were excluded not being related to the research topic/research aims or questions. Twenty-five studies were scrutinized in the full text. Out of these 25 items, 8 studies [16,17,18,19,20,21,22,23] were excluded with reason (n = 2, not reporting sufficient quantitative/qualitative details; n = 4, not disaggregating data according to para-sports discipline; n = 2 reporting details of another study (letter and response to the editor). Finally, 17 studies [5,6,7,24,25,26,27,28,29,30,31,32,33,34,35,36,37] were retained in the present scoping review. The included studies were found to focus on a range of aspects involving health, the etiology of injuries (n = 5), psychological and physiological responses (n = 5), performance, biomechanical analysis (n = 4), and new analytical approaches for kinematic assessments and predictions of mechanical outputs in para-rowers (n = 3). The flow-chart adopted in the present scoping review is pictorially depicted in Figure 1.

### 3.2. Psychological and Physiological Responses in Para-Rowing

This section included five studies [24,25,26,27,28], which are overviewed in Table 1. Of these, four studies [24,25,26,27] investigated physiological responses to para-rowing. Only one study [28], on the other hand, investigated the psychological aspects.

Zoppi et al. [24] examined changes in anthropometric (body mass and skinfold), physiological (muscle strength, power at 4 mM blood lactate, and fatigue index), and performance (1000 m trial) parameters throughout 32 weeks of training in a sample of eight Afro-Brazilian para-rowers (aged 25 ± 5.3 years). Four participants had a unilateral traumatic above-the-knee amputation, one subject had a unilateral hip disarticulation, one individual had Class 5 cerebral palsy, one subject had Class 4 cerebral palsy, and one subject had a neurological impairment (a complete lesion at the L3 level). The training protocol was designed in 4 phases (phase 1 or incorporation phase: 6 weeks, phase 2 or basic phase: 6 weeks, phase 3 or specific phase: 12 weeks, and phase 4 or competitive phase: 8 weeks), which were different in terms of volume and intensity, intending to achieve peak form in the last phase. The studied anthropometric characteristics remained stable during the initial phases of training with a significant drop in body mass in the penultimate (*p* ≤ 0.05) and last phase (*p* ≤ 0.1) and in body fat in the last phase (*p* ≤ 0.05). Regarding physiological parameters, muscle strength significantly improved in all three trials performed (lying T-bar row, barbell bench press, and 45° leg press with one repetition max) after phase 3, with a peak in phase 4 (*p* < 0.01) In addition, during maximal intensity exercise (1000 m trials), power at 4 mM blood lactate and fatigue index improved in all phases and in the penultimate and last phase, respectively (*p* < 0.01). However, no significant variation in power output was found. Finally, performance improved immediately after phases 1 and 2 (*p* < 0.01 compared to the start of training) with a peak after phase 3, which was maintained until the end of phase 4 (*p* < 0.01 compared to the start of training and to phases 1 and 2).

To understand whether high training loads lead to higher levels of fitness, Porto et al. [25] compared the physiological (aerobic, anaerobic, strength levels, and fatigue index) and anthropometric (body mass and body fat) parameters of eight top para-rowers (aged 30 ± 9.25 years) matched with a control group of eight wheelchair basketball players (aged 45.4 ± 9.6 years) who did not practice regularly. Furthermore, to better understand how motor disabilities compromise performance in the para-rower group, a comparison with literature data concerning high-level able-bodied rowers was conducted on the studied parameters. The authors showed that para-rowers tended to have similar strength levels and fatigue, but a higher anaerobic threshold (*p* < 0.01) than the control disabled group. However, among all the anaerobic performance outcomes studied, only the peak anaerobic weight/power ratio was significantly higher (6.5 ± 1.1 W/Kg vs. 7.5 ± 0.9 W/Kg, *p* = 0.0387). Regarding the anthropometric parameters, the two groups of people with disabilities had similar body mass, but the para-rowers had significantly lower body fat. Compared with high-level able-bodied rowers, the para-rowers showed ten times greater fatigue levels and a 37% lower anaerobic threshold with lower anaerobic performance capacity. Specifically, mean, and lower anaerobic power were 26% and 54% lower, respectively. Contrary to all the trends, the weight/peak anaerobic power ratio was 25% higher in para-rowers. 

While the physiological response and adaption to exercise have been highly studied in able-bodied athletes, there is a significant dearth of data concerning post-exercise changes in athletes with disabilities.

In this regard, Nowak et al. [26] recruited two para-rowers from a Polish adaptive rowing settle TAMix2x qualified for the Paralympic Games in Rio, 2016. The authors conducted a progressive test on a rowing ergometer until exhaustion, assessing a range of parameters including cardiorespiratory fitness, complete blood count, white blood cell distribution, and thirty clinical chemistry variables. Exercise-induced changes could be detected for all metabolites under study (glucose, creatinine, urea, uric acid, and both total and direct bilirubin), as well as for albumin, total protein levels, aspartate transaminase activity, and white blood cell count. Concerning the latter two variables, a post-exercise increase was found for both parameters, but with a different post-recovery response, characterized by a two-fold decrease and increase, respectively. The percentages of natural killer cells and total T lymphocytes were found to be higher and lower after the exercise, respectively. Of interest, although changes in T lymphocyte subset distribution could be noted (with higher and lower percentages of suppressor/cytotoxic and helper/inducer cells), no changes in B lymphocyte distribution could be observed.

Tiller et al. [27] conducted a case study to evaluate diaphragm fatigue (measuring pre- and post-exercise changes in the transdiaphragmatic pressure response of contraction to anterolateral magnetic stimulation of the phrenic nerves) in a spinal cord injured para-rower (aged 28 years old) during a test on a rowing ergometer (1000 m). In addition, pulmonary ventilation and gas exchange, diaphragm electromyography (EMG)-derived indices of neural respiratory drive, and respiratory mechanics indices derived from intrathoracic pressure were evaluated. The para-rower completed the test in 3.89 min at an average power of 248 W, with peak oxygen uptake and pulmonary ventilation of 3.46 L/min and 150 L/min, respectively. The blood lactate concentration reached 15.8 mmol/L (8 min after exercise). The breath/stroke ratio was 1:1 in the initial part of the test (0–400 m). Subsequently (after 400 m), a reduction in respiratory time and an increase in ventilatory drive resulted in a breath/stroke ratio of 2:1. From baseline, the reduction in transdiaphragmatic pressure at 15–20 min after exercise was 33% (61 vs. 41 cm H_2_O) with a partial recovery of 12% at 30–35 min (41 vs. 50 cm H_2_O) (10–15% reduction is already considered muscle fatigue). The muscle fatigue that occurred was associated with a reduction in the neuromuscular efficiency of the diaphragm. Specifically, the inspiratory transdiaphragmatic pressure decreased throughout the test, whereas the EMG value tended to increase (16.0 vs. 3.0 as the ratio of the two outcome measures studied). Finally, after the test, due to an increase in tidal volume (from 2.7 to 3.1 L), an increase in absolute ventilation was found (from 13.6 to 18.7 L), whereas the transdiaphragmatic pressure and the EMG of the diaphragm decreased (133 to 53 cmH_2_O; 91 to 58% of the maximal value of the Root Mean Square, RMS_max_).

In addition, Rich et al. [28] aimed to capture elite para-rowers’ embodied experiences with their engagement in sport, in terms of benefits and challenges, barriers, and facilitators, by conducting an interpretative phenomenological analysis. The authors recruited eight participants with a visual impairment aged 40.5 ± 16.3 years old (22–66), representing three countries: seven of them were female, and one was male. Five of the participants’ visual impairments were congenital. According to the USABA Visual Impairment Classification System, four participants reported having B3 vision (low vision), two reported having B2 vision (travel vision), and two reported having B1 vision (blind). The authors were able to extract four major themes: namely, (i) empowerment through para-rowing, (ii) rowing through feel, (iii) changing perceptions, and (iv) establishing influential, supportive, inclusive, and meaningful relationships. Para-rowing allowed them to overcome fears related to their impairment and cope with their acuity/vision loss, abate apprehensions, gain independence and psychological benefits, including resilience, self-esteem, confidence, and self-advocacy, and generate opportunities as well as social connections, relationships, and networking, in terms of shared commonalities and mutual support. Para-rowers were also able to better manage their time and build self-discipline, set, and achieve new goals. Finally, para-rowing was considered more accessible for people with visual impairments than other Paralympic sports disciplines, being “based on feel, regardless of sight” and given that no additional equipment and special accommodations/modifications were necessary for rowing and racing with a visual impairment. On the other hand, participants also experienced negative feelings and emotions, due to ableism derived from ignorance and the stigma of visual impairment: para-rowers had to make an effort to either formally or informally educate their coaches and peers toward acceptance of their disability. Para-rowing was perceived as a second-class and less legitimate sport in comparison with rowing, and para-rowers’ athleticism, and achievements were underscored with respect to their able-bodied counterparts.

### 3.3. Health, Injuries, and Risk Factors in Para-Rowing 

Five studies [5,7,29,30,31] explored the determinants of health and injuries, including their risk factors, in para-rowing. Studies included are briefly overviewed in Table 2. 

Smolyanović et al. [7] conducted a case study on the management of a rib stress fracture in a PR1 rower (23 years old) caused by pressure occurring in the chest strap area during hyperflexion of the torso in the catch position. Two different strategies were followed by the coaching staff: (1) pause from activity until the athlete no longer felt tenderness on palpation and deep inspiration (5 weeks) (2) the use of a protective orthosis to relieve the pressure of the chest strap on the chest has been studied and applied. However, the para-rower during the forced stop was unable to maintain the specific physical form of rowing, compromising his athletic preparation and giving up participating in a world championship.

Soo Hoo et al. [29] conducted a retrospective survey of non-elite athletes with disabilities to assess their demographics, training regimen, and injuries. Of the total of 61 athletes approached, 43 athletes participated in the study and five of whom practiced rowing (4 females and 1 male). The main and extremely interesting finding was that none of the rowers were injured in the last 12 months, whereas around half of participants in sled hockey (50%), wheelchair basketball (44%) and wheelchair rugby (43%) suffered an injury. Data on training regimens, on the other hand, are in line with those of rowers. Specifically, most athletes trained in their sport almost all year round with an average of about 8 h per week. Regarding the diagnosis, spinal cord injury was the most frequent (51%) and was predominant in rowers (100%). Finally, athletes with disabilities involved in rowing and basketball had a higher incidence of spasticity (80% and 100%, respectively) than the overall average (42%).

As of 2017, the race distance for para-rowers has changed from 1000 m to 2000 m for all events. The review by Smoljanović et al. [5] analyzes the potential injuries and health risks associated with this regulation change in PR1 and PR2 rowers. The authors find no significant evidence that increasing distance and consequently race time leads to more problems. However, it is recommended to modify the training program in order to prepare the athletes for the doubled distance.

Thornton et al.’s review [30] analyzed the risk factors of disability-dependent injuries. For example, a para-rower with a leg strength deficit will compensate more with the other leading to imbalances in the entire kinetic chain and contributing to possible lower back injuries. Furthermore, the skin lesion of the stump caused by the prosthesis in the amputee athlete can compromise athletic preparation until the skin heals. Additionally, special attention should be paid to athletes with spinal cord injuries. Athletes with this condition may be at increased risk for bone fractures and/or joint dislocations and pressure sores. The latter problem can cause an increase in muscle spasticity and in case of severe infection even death. Finally, the chest straps create high pressure on the athlete’s chest, placing them at risk for rib stress fractures.

Nowak et al. [31] performed a comparative study, assessing the degree of adoption of healthy lifestyles in people with disabilities, using wheelchairs in their daily lives, and practicing wheelchair basketball, wheelchair rugby, and para-rowing. The sample consisted of 176 participants, aged 19–49 years old (mean age 34.41 ± 8.56 years), mostly under 40, from all over Poland, men (83.5%), city-dwellers, in formal relationships, with higher education, working professionally, and assessing their financial situation as good. 35 were para-rowing athletes. Compared with the average participants, they were more often unmarried, lived off their pension, and rated their finance lower. 42.9% of them won the title of the Polish Champions. With respect to the others, they reported a lower intensity of health behaviors in general and in four categories of correct eating habits, preventive behaviors, positive mental attitude, and health practices. Compared with Polish Champions, amateur rowers with disabilities achieved the poorest results in terms of preventive behaviors but the highest results in positive mental attitudes. Training every day and having the longest weekly exercise, having received higher education, being in a better financial situation, being employed, married, and being rural residents correlated with a greater intensity of health-related behaviors.

### 3.4. Biomechanics of the Para-Rowing Strokes

Four studies [6,32,33,34] provided insight into the biomechanics of rowing in the Paralympic setup. These are briefly overviewed in Table 3.

In one case study, Schaffert and Mattes [32] used biomechanical measurements combined with questionnaires to examine the effects of acoustic feedback on mean boat speed in seven training sessions of the same rowing crew (coxed four) with visual impairment (aged 34.8 ± 10.6 years). The training consisted of five blocks of 500 m at two different intensities (20 and 22 rpm). In both intensities, in the first, third, and fifth blocks, acoustic feedback is provided, whereas in the second and fourth blocks it is not. Furthermore, a baseline was obtained once for each intensity. The authors found that the three blocks with acoustic feedback for both intensities were faster than the baseline (0.08 ± 0.01 m·s^−1^; *p* < 0.01). In the highest intensity test (22 rpm), a different speed was found between blocks with acoustic feedback. Specifically, the first block was slower than the third (0.08 ± 0.01 m·s^−1^; *p* = 0.001; effect size, ES = 1.11) and the fifth (0.07 ± 0.01 m·s^−1^; *p* = 0.001; ES = 0.93). Furthermore, the blocks without acoustic feedback of both intensities (second and fourth blocks) were faster than baseline but slower than with acoustic feedback. From the analysis of the time structure of the rowing cycle, the acoustic feedback would seem capable of optimizing the time course of boat acceleration, mainly during the recovery phase, reducing its fluctuations and thus increasing the speed. Finally, the positive effects of acoustic feedback are also confirmed by both athletes and coaches (by completing a questionnaire) who generally perceive a better smoothness of the movement performed during the recovery phase compared to without acoustic feedback.

Held et al. [33] compared kinematic and physiological data and performance during tasks performed with two different boat configurations. Specifically, this was carried out with a standard setup (NORM: with the oar in front of the axis of rotation) vs. a modified setup (GATE: with the oar behind the axis of rotation) in a sample of 15 able-bodied rowers and a PR1 rower. The able-bodied and the para-rower performed, respectively 2 and 24 timed trials of 2 min each. The former was in an indoor rowing tank, whereas the latter was in the water. Both performed the time trials in both NORM and GATE setups in randomized order at maximum intensity with legs and trunk fixed with straps and with a fixed stroke rate (34 rpm). The result shared by all participants indicates that GATE (compared to NORM) enables larger catch angles. Specifically, for this output parameter, there was a mean increase of 97% (*p* < 0.001) and 12% (*p* = 0.021) for able-bodied athletes and for para-athletes, respectively. However, no changes in the shape of the force-angle curve (position of peak force and ratio of mean to maximum force) were found. Furthermore, with GATE, able-bodied athletes experience an increase in total stroke length (*p* < 0.010), rowing power (55.8 ± 57.3%, *p* < 0.010), and work per stroke (59.7 ± 67.2%, *p* < 0.010). In the Paralympic athlete, on the other hand, these performance parameters are unchanged. Concerning the specific parameters of the para-rower, the economy of the rowing (power or speed per oxygen uptake) and the speed of the boat did not show significant differences between the two setups used.

Cutler et al. [34] investigated how para-rowing configurations change physiological measures (handle pull force), kinematics (stroke length), and posture (grip/landing angles on specific joints) in ergometer rowing. In total, 17 able-bodied rowers (9 men and 8 women) completed three 10-stroke trials in each rowing configuration (PR1, PR2, and PR3). Physiological and kinematic parameters in the PR3 configurations were comparable to values for the able-bodied literature and decreased with configurations. Specifically, handle force decreased from PR3 to PR2 set-up by 22% and from PR2 to PR1 by 42%. Similarly, stroke length decreased with the PR3 to PR2 setting by 25% and PR3 to PR1 by 47%. For both parameters, men performed better than women. Regarding posture in the PR 2 setups, the rowers used a greater lumbar angle at the catch (37° vs. 29°) and at the finish (−42° vs. −39°) of the stroke compared to the PR3 setups. On the other hand, the flexion of the elbow joint at the finish of the stroke was 11° and 7° greater, respectively, in the PR1 setup compared to PR3 and PR2. Finally, shoulder abduction at the finish of the stroke increased from LTA to TA (*p* = 0.014), LTA to AS (*p* = 0.001), and TA to AS (*p* = 0.006) configurations.

In a case study, Severin et al. [6], compared the effects of a more inclined seat and backrest on rowing performance, physiology, and biomechanics parameters in a PR1 world champion woman athlete (aged 30 years old). The test protocol consisted of three 4 min phases performed with a target power of 100 W (SUBMAX test) and an all-out effort (MAX test) in three different seat configurations (conA: 25° from the vertical plane and 7.5° from the horizontal, conB: 25° from the vertical plane and 0◦ from that to the horizontal, conC: 5° from the vertical plane and 0° from the horizontal plane). During SUBMAX due to the nature of the task (target power 100 W) similar virtual distances were recorded (conA: 793, conB: 793, conC: 787 m). However, in the usual setups (conC), the peak force (conA: 509, conB: 458, conC: 312 N) and impulse (conA: 172, conB:158, conC: 97 N∙s) were lower as well as the stroke length (conA: 81, conB: 78, conC: 67 cm) compared to the other two setups. To compensate for this result, there was an increase in stroke rate (conA: 27, conB: 31, conC: 49 strokes·min^−1^) at the expense of increase in VO_2_ (conA: 34.4, conB: 35.4, conC: 39.6 mL·kg^−1^·min^−1^). Similarly, in the MAX task, the conC configuration was less performing in terms of distance covered (conA: 934, conB: 918, (conC: 856 M), peak force (conA: 408 N, conB: 418 N, conC: 331 N), stroke length (conA: 79, conB: 77, conC: 68 cm), and stroke rate (conA: 51, conB: 54, conC: 56 rpm). However, these differences were smaller than in SUBMAX test. Regarding the kinematic parameters, the para-athlete was able to perform a greater extension of the trunk in setups conA and conB compared to conC. Finally, an increase in flexion in the elbow and shoulder joints in the SUBMAX task during the driving phase was found in conA and conB compared with conC.

### 3.5. Emerging Analytical Approaches for Kinematic Assessments and Predictions of Mechanical Outputs in Para-Rowers

Three studies [35,36,37] developed new analytical approaches for kinematics assessments and predictions of mechanical outputs in para-rowers. These studies are overviewed in Table 4.

In a multi-time assessment (longitudinal search), it is critical that the test chosen can consistently reproduce the same outcome between visits, provided all other variables remain the same. In this regard, Euiler and Finley [35] assessed the repeatability of the para-rowing stroke and analyzed EMG data (muscle activity of the upper, middle, and lower trapezius, anterior and posterior deltoid, latissimus dorsi, and infraspinatus) and kinematic of the rowing stroke (humero-thoracic plane of elevation, angle of elevation, and trunk flexion and extension, and trunk rotation) in a sample of 10 para-rowers (7 males and 3 females aged, 41.6 ± 13.4 years old) with low experience, to determine the trial-to-trial reliability of three submaximal rowing trials (20 strokes each). Muscle activity and kinematics data were reliable with moderate to excellent and excellent intraclass correlation coefficients, respectively.

Schwingel et al. [36] conducted a study with a dual objective: to analyze the safety and tolerability of performing the one-repetition maximum tests (1RM) and compare the 1RM–measured values with linear and exponential equation models (12 different equations) for predicting 1RM. Two upper body strength exercises (Flat barbell bench press and Lying T-bar row) and one lower body (45-degree leg press) were performed by 9 male para-rowers (mean age; 30 6 7.9 years, 7 one-legged amputees and 2 with cerebral palsy). From qualitative analysis, the 1RM test proved to be well-tolerated and safe also for athletes with a disability. However, for body strength exercises, the estimate of maximal strength can be replaced by prediction equations. In fact, although the predicted values were slightly underestimated, they were accurate and reliable. Furthermore, the authors did not find statistical differences between exercises (*p* = 0.84 and 0.23 for lying T-bar row and flat barbell bench press, respectively). For lower body exercise, on the other hand, a highly significant difference between measured and predicted values were found (*p* < 0.01).

People affected with upper motor neuron syndrome may benefit from functional electrical stimulation (FES) through the electrical elicitation of paralyzed muscles [22]. FES has been integrated with rowing (FES Rowing) also allowing people with paraplegia to row without adapting to boat configurations [20]. To maximize the benefits of FES Rowing, the intensity of a muscle’s stimulation should be greatest when its force-producing potential is greatest. This is possible by monitoring the joint angles of the specific muscle during rowing. Concerning that point, Vieira et al. [37] compared a biomechanical model for knee joint angle estimation with actual knee angle changes (measured with inertial sensors) during three sets of 30 strokes (each at a different stroke rate of 18, 24, and 32 spm) in indoor rowing in a sample of 15 able-bodied (age range 20–35 years) and 11 PR3 rowers (23–47 years). This model was based on real-time measurements of handle and seat position for each stroke transmitted by an adapted rowing machine. The authors found that the mean squared error (RMSE) between calculated vs. estimated was generally low (3.8 to 5.1 degrees). Furthermore, the estimation error differed significantly within the rowing cycle (*p* < 0.001) and not for group and stroke rate (*p* > 0.267). Specifically, the highest RMSE values occurred during 20–50% and 80–100% of the rowing cycle (*p* < 0.003) with a peak during the mid-recovery (average 8 deg). Additionally, for the two groups and for the three-stroke rates, the onset of knee flexion was consistently underestimated (∼5%, *p* < 0.001). Based on these results, the biomechanical model used proved to be reliable and capable of replacing the less economical inertial sensors for kinematic analysis.

### 3.6. Bibliometrics-Based Analysis of Para-Rowing Scientific Output

**Figure 2:**
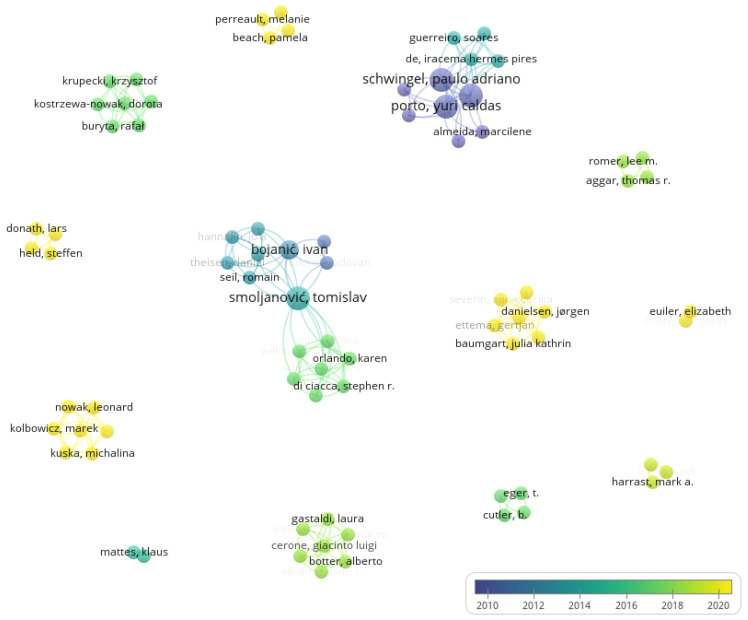
Bibliometric map showing connections of the authors in the field of para-rowing.

The bibliometrics analysis enabled us to identify 78 researchers (nodes), 16 (20.51%) of whom were highly interconnected (Figure 2). The resulting graph (Figure 2) consisted of 202 links (edges), with a total link strength of 209, and 14 clusters. The most prolific author was Smoljanović, T., with 3 items/documents (representing 17.6% of the scientific output overviewed in the present scoping review). The list of the ten most productive scholars can be found in Table 1. In total, 93.6% of scholars have authored one single document. The main topological features of the scholarly community of authors on PR are shown in Table 5 and Table 6: these features indicate a highly fragmented and dispersed, poorly connected community characterized by a relatively high number of clusters and a low strength of connections. In terms of publication years, the first scholarly article dates back to 2008, with four articles (23.5%) published in the current year, showing an increasing interest in this para-sports discipline.

The top institution was the University of Zagreb, Zagreb (Croatia). In terms of journals, the studies included in our review were published in 15 scholarly journals, in the field of sports science, orthopedics and sports medicine, physical therapy, sports therapy, and rehabilitation, medicine (miscellaneous), physiology and medical physiology, rehabilitation, public health, environmental, and occupational health, biomedical engineering, computer science applications, internal medicine, neuroscience (miscellaneous), biophysics, and neurology/neurology (clinical). The two top journals were the International Journal of Environmental Research and Public Health (IJERPH) and the Journal of Sports Sciences. More than half of the journals (53.3%) were top journals in their fields. More in detail, four journals (namely, the British Journal of Sports Medicine, the Journal of Strength and Conditioning Research, Frontiers in Physiology, and the Journal of Sports Sciences) were first quartile (Q1) journals, whereas four other journals (IJERPH, the Journal of Applied Physiology, the Journal of Human Kinetics, Research in Sports Medicine, and IEEE Transactions on Neural Systems and Rehabilitation Engineering) were Q1-second quartile (Q2) scholarly journals. In terms of citations, the most cited article was the study by Thornton et al. [30], with 36 citations, according to Scopus. Finally, in terms of funding, this was reported by six articles: more specifically, several sponsors/funding sources could be identified, including universities and research centers, research councils and foundations, and Ministries/governmental agencies and institutions.

## 4. Discussion

### 4.1. Psychological and Physiological Responses in Para-Rowing

Due to the nature of their disabilities, para-rowers have exhibited lower fitness levels than their able-bodied counterparts [25]. Indeed, some disabilities limit the physiological functioning of these athletes. For example, spinal cord injuries, which are the most commonly reported type of impairment in para-rowers, can lead to impaired cardiovascular and respiratory function, characterized by decreased peak heart rate, inability to increase tidal volume, exercise-induced hypotension, and increased fatigue [27,38]. In addition, decreases in strength (impaired neural recruitment), and ROM, as well as increased muscle stiffness, spasticity, spastic dystonia, and impaired coordination, have been found in athletes with cerebral palsy [39,40]. However, from the studies retained in the present scoping review, two important and highly interconnected aspects emerged, stating the beneficial effect of training. The first aspect is that para-rowers tolerate the training load of able-bodied rowers relatively well, allowing for increases in performance-outcomes aspects such as muscle strength, anaerobic power, and aerobic power [24]. The second is that with the right training program, para-rowers did not show any inflammatory symptoms, indicating high levels of immunological adaption [26]. Regarding the psychological aspects, para-rowing is an empowering, engaging, and accessible sports discipline that can provide athletes with meaningful connections and social networking, enabling them to overcome stigma and disability-related fears and apprehensions [28].

### 4.2. Health, Injuries, and Risk Factors in Para-Rowing Injuries

The rowing of an able-bodied rower represents a sequence of movements that are part of a single kinetic chain in which the trunk acts as a link generating and transferring forces from the legs and arms to the oar [4,41]. In para-rowers, this is not the case due to the limited joints and body segments used. This results in isolated repetitive force generation of joints and functional segments and an increased risk of injury [7,30]. In addition, increasing stroke rate, boat weight and racing time add further stress to the musculoskeletal system [5]. The initial-to-mid drive phase is particularly critical in the PR1 and PR2 sports classes. In fact, to reach the maximal stroke length, in these phases they perform larger amplitudes of forward flexion of the trunk (in the PR2 class) and shoulder flexion (in PR1 and PR2 classes) in comparison to PR3 class and/or able-bodied rowers, which can be potentially harmful [34]. Additionally, PR1 rowers with minimal trunk control bend as much as possible at the chest strap, creating stress on the upper thoracic region with a high risk of rib fractures [7]. However, para-rowing compared to other Paralympic sports such as sled hockey, wheelchair basketball, and wheelchair rugby would seem the safest [29]. Finally, most rowers in class PR1 and PR2 have a spinal cord injury. The former generally have lesions in the thoracic spine while the latter have lesions in the lower thoracic or lumbosacral spine [22]. It is, therefore, conceivable that the greater risk of injury within these sports classes is closely connected to the underlying pathology. For example, lesions attributable to autonomic dysreflexia, spastic hypertonia and osteoporosis (such as bone fractures resulting from small traumas) [30].

### 4.3. Biomechanics of the Para-Rowing Strokes

The biomechanics of the para-rower is significantly altered mainly due to the pathology. Equipment adjustments made to minimize intra-class differences can make this situation worse. In fact, it has been observed that rowing with a PR2 setup or even more with a PR1 setup alters the range of motion and measures of handle strength and stroke length even for able-bodied athletes [34]. In this regard, various strategies have been studied to implement the para-rowing set-ups, some of which have proved to be useful, while others have proved to be difficult to transfer to the para-rowers. For example, because of the importance of allowing trunk extension in para-rowers with minimal residual trunk function, a more inclined seat and backrest were used [6]. This modification allowed a more trunk motion and a longer stroke length combined with a lower stroke rate. In fact, moving the oar from in front of the axis of rotation to behind the axis of rotation instead made biomechanical improvements primarily for able-bodied athletes [33]. This confirms that applying the discoveries made in the world of able-bodied to the para-athlete populations could be a mistake. The latter is a widely heterogeneous world with intra- and interdependent implications of the specific type of impairment. Finally, an acoustic feedback strategy was studied for athletes with visual impairment during training sessions [32]. Acoustic feedback has proved to be useful for optimizing the time course of boat acceleration during the rowing phase (mainly during the recovery phase). Specifically, the para-rowers performed a more regular sliding movement in the recovery phase compared to the situation without feedback, increasing the force output in the drive phase and consequentially the speed of the boat [16,21].

### 4.4. Emerging Analytical Approaches for Kinematic Assessments and Predictions of Mechanical Outputs in Para-Rowers

Having reliable, validated measures is of paramount importance in the field of research. Para-rowing stroke-related metrics have shown excellent repeatability [35]. The 1RM test is relatively safe and easy to evaluate and correlates well with sports performance [42]. However, pulling to the maximum implies maximum stress for the involved structures, such as muscles, tendons, joints, and even neural networks. For this reason, in para-athletes, the 1RM test is often replaced by alternative tests to prevent injuries, technical failures, and fatigue [43,44,45]. While in the upper limb both methods (i.e., 1RM and alternative tests) can be used to assess the intensity of resistance training, in the lower limb 1RM is more accurate than alternative tests, especially for athletes with one leg amputated [36]. Besides strength assessment, biomechanical evaluation is also fundamental. The latter is usually carried out by using inertial sensors, which are generally expensive and require calibration efforts, which is rather a time- and resource-consuming procedure [46]. A study developed a significantly less expensive and similarly reliable methodology, that can be easily implemented in the setting of para-rowing. More specifically, the adaptation of the rowing machine enabled the provision of real-time data on handle and seat position, that a subject-specific biomechanical model was able to relate to the knee joint angle [37].

### 4.5. Bibliometrics-Based Analysis of Para-Rowing Scientific Output

A few bibliometrics studies have investigated scholarly interest in Paralympic disciplines. Of interest, the most over-represented countries such as Croatia, Brazil, and Canada, did not qualify as top counties in terms of medals awarded. Recently, our group assessed publishing trends related to Paralympic powerlifting [15]. Compared with the Paralympic powerlifting community, the para-rowing community appears to be less connected, more fragmented, and dispersed. Of note, no hub authors could be identified, and the number of documents/items are more homogeneously distributed among authors, with the most prolific scholar having coauthored three studies. For para-rowing, Canada was over-represented as a country, while Brazil was over-represented for Paralympic powerlifting. Finally, also in terms of temporal trends, publication years were more homogeneously represented and distributed from 2008 to 2022. In the sports arena, scientific collaboration is of paramount importance in that it can enhance the quality of research, by improving performance-related outcomes, establishing networks, and achieving more sustainable development. This is even more true in the Paralympic world, where the complexity of the issues makes collaborations vital for the advancement of knowledge. Therefore, further efforts are warranted to improve the scientific collaboration among authors interested in para-rowing.

### 4.6. Gaps in Knowledge and Future Directions

Overall, a relative paucity of evidence-based studies was identified, particularly in the domain of physiology, probably due, at least partially, to the recent debut of para-rowing at the Paralympic Games. The presence of mostly case studies, anecdotal reports, articles based on personal experiences, and/or investigations mainly focusing on a single type of impairment makes it difficult to have a comprehensive understanding of how various impairments could affect the physiological response to exercise and adapted rowing. Accurate, rigorous knowledge of these aspects could serve a two-fold role: (i) to preserve, and, potentially, enhance, the health and well-being of the para-athlete, and (ii) to plan individualized interventions, and devise specific protocols, conditioning, and training methods, which are appropriate based on the specific type of disability. For example, it can be anticipated that athletes with visual and intellectual disabilities show very different physiological responses to exercise than athletes with spinal cord injuries. Additionally, there could be a significant degree of variability among athletes with the same impairment. For instance, in subjects paralyzed with spinal cord injuries, disability depends on the level at which spinal cord is injured and varies whether the impairment is congenital or acquired [47,48]. 

To ensure fair competition, athletes with similar levels of activity limitation due to their impairments should be in the same sports class and compete against each other. However, one of the major limitations of para-rowing is a lack of evidence-based classification to assess the true impact of impairment on performance. Furthermore, a broad range of disabilities and just three sports classes (PR1, 2 and 3) might give some athletes an advantage over others. Therefore, it is necessary to develop tests that describe impairment and define the relationship between impairment and rowing performance outcomes. In other words, a correct classification method should be based on scientific evidence and not on expert opinions. 

The use of technology to measure motor disability has proven useful in several Paralympic sports [49] and could also be used in para-rowing. For example, para-swimming technology has enabled more objective and reliable tests to be designed which are likely to be included in the next revision of the World Para Swimming classification [50,51,52]. Moreover, also as far as injury is concerned, data is still lacking in this athlete population [30]. In most rowing injury cases there is a history of inaccurate management of load parameters with increased intensity/length of training associated with poor recovery [7]. However, training methodology and periodization theory of peak performance represent relatively under-explored and overlooked research areas in the existing academic literature. Additionally, to our knowledge, training methods for maintaining conditioning during periods of inactivity have never been explored. 

The limited body of evidence can lead the less expert coach to manage the para-athlete as an able-bodied athlete, compromising their preparation and, in the worst case, their health. While kinematic evaluations have been extensively studied, topics such as sports nutrition, doping, and psychology have been relatively underdeveloped and overlooked. Psychological aspects have been investigated only in athletes with loss of visual acuity, who do not represent the most commonly described para-rower, usually affected by spinal cord injury.

Therefore, in para-rowing, further studies are warranted involving a broad range of impairments and disabilities, with particular attention paid to spinal cord injury. Finally, although in recent years COVID-19 has dramatically impacted the Paralympic sport arena [53], there are no published studies investigating this topic, which deserves to be explored.

## 5. Conclusions

The current literature review covered several domains and aspects of para-rowing, namely (i) psychological and physiological aspects and responses in para-rowing, (ii) epidemiology and risk factors of para-rowing injuries, (iii) biomechanics of the para-rowing strokes, and (iv) emerging analytical approaches for kinematic assessments and prediction of mechanical outputs in para-rowers. This literature review was complemented by a bibliometrics-based analysis of para-rowing scientific output. This information can be used by both practitioners and athletes, to enhance their expertise and knowledge of training protocols and conditioning strategies. However, several gaps were identified in terms of underdeveloped or overlooked topics (namely, sports nutrition, doping, and psychological aspects in para-rowers other than those with visual impairment), warranting further research in the field. 

## Figures and Tables

**Table 1:** Main characteristics of the study included in the present scoping review concerning psychological and physiological responses in para-rowing. Abbreviations: BF (body fat); BM (body mass); EMG (electromyography); FI (fatigue index); IF (Impact Factor); NA (not available); SCI (spinal cord injury); w (weeks); y (years).

Study, Study Design	Journal, IF (2022), Scimago Ranking, Subject Areas and Categories	Sample Size, Sex, Age, Experience, Type of Disability	Sports Class	Aim(s)	Outcome Measures	Findings
Zoppi et al., 2014 [24]; Longitudinal study	Journal of Exercise Physiology Online, NA, Q4; Medical Phsyiology	8; Male; 25 ± 5.3 y; International level; Physical disability	3 LTAMix4+, 3 TAMix2x, 2 ASM1x	Evaluation of physiological and performance changes during a training season (32 w)	BM, muscular strength, power at 4 mM blood lactate, FI, and a 1000 m time-trial	Outcomes improved
Porto et al., 2008; [25]; Comparative (case–control) study	Research in Sports Medicine; 3.661; Q1–Q2; Medicine (miscellaneous); orthopedics and sports medicine; physical trherapy, sports therapy and rehabilitation; sports science	8; Male; 30 ± 9.25 y; International level; Physical disability	NA	Evaluation of anthropometric and physical characteristics of motor disabled para-rowers	Upper body anaerobic threshold, peak, mean, and lower anaerobic power, peak anaerobic power to weight ratio, FI, BF, handgrip strength	Similar strength levels and fatigue, but a higher anaerobic threshold and peak anaerobic weight/power ratio, similar BM, but lower BF with respect to disabled controls; higher FI and lower anaerobic threshold with lower anaerobic performance capacity and higher weight/peak anaerobic power ratio with respect to able-bodied controls
Nowak et al., 2017 [26]; Case series	Journal of Human Kinetics; 2.923; Q1–Q2, Physical therapy, sports therapy and rehabilitation; medical physiology; sports science	2; 1 male, 1 female; 26 and 38 y; from 6 to 10 y of training experience; Physical disability	TAMix2x	Evalution of the impact of the Progressive Efficiency Test on a rowing ergometer on clinical chemistry parameters	Cardiorespiratory fitness, blood count, white blood cell distribution, and 30 clinical chemistry variables	Changes in study variables, with a few exceptions (no changes in B lymphocyte distribution)
Tiller et al., 2018 [27]; Case report	Journal of Applied Physiology; 3.880; Q1–Q2; Medicine (miscellaneous); Physiology and medical physiology; Sports science	1; Male; 28 y; Elite; SCI	ASM1x	Evaluation of diaphragm fatigue on a rowing ergometer (1000 m)	Pulmonary ventilation and gas exchange, diaphragm EMG-derived indexes of neural respiratory drive, and intrathoracic pressure-derived indexes of respiratory mechanics	Decrease in diaphragm neuromuscular efficiency during exercise
Rich et al., 2022 [28]; Interpretative Phenomenological Analysis	International Journal of Environmental Research and Public Health; 4.614; Q2; Public health, environmental and occupational health	8; 7 female and 1 male; 40.5 ± 16.3 y (22–66); Elite; Visual impairment (4 B3, 2 B2, 2 B1)	NA	To capture elite para-rowers’ embodied experiences with their engagement in sport	Benefits and challenges, barriers, and facilitators	Empowerment through para-rowing, rowing through feel, changing perceptions, and establishing influential, supportive, inclusive, and meaningful relationships.

**Table 2:** Main characteristics of the study included in the present scoping review concerning health, injuries, and risk factors in para-rowing. Abbreviations: IF (Impact Factor); NA (not available); SCI (spinal cord injury); y (years).

Study, Study Design	Journal, IF (2022), Scimago Ranking, Subject Areas and Categories	Sample Size, Sex, Age, Experience, Type of Disability	Sports Class	Aim(s)	Outcome Measures	Findings
Smoljanović et al., 2013 [5]; Review study	British Journal of Sports Medicine; 18.473; Q1; Medicine (miscellaneous); orthopedics and sports medicine; physical therapy, sports therapy and rehabilitation; sports science	NA	NA	To evaluate the potential injuries and health risks associated with regulation changes in para-rowing	NA	No potential health risks
Smolyanović et al., 2011 [7]; Case study	Croatian Medical Journal; 2.415; Q3; Medicine (miscellaneous)	1; male; 23 y; <1 y of experience training; physical disability (SCI, T9 complete paraplegia)	PR1	To evaluate the management of a rib stress fracture	NA	The athletic preparation was compromised and the athlete had to give up participating in a world championship
Soo Hoo et al., 2019 [29]; Retrospective survey	Journal of Injury, Function and Rehabilitation; 2.218; Q1–Q2–Q3; Medicine (miscellaneous); neurology and clinical neurology; physical therapy, sports therapy and rehabilitation; rehabilitation; sports science	5; 4 females, 1 male; 3 30–40 y; Non-elite; Physical disability	NA	To evaluate and assess the demographics, training regimen, and injury rate in para-rowing	Self-report data	No injuries reported in para-rowers. Half of the participants in other sports had been injured. Training regimen was 8 h per week. SCI was the most common disability
Thornton et al., 2017 [30]; Review study	Sports medicine; 11.928; Q1; Medicine (miscellaneous); orthopedics and sports medicine; physical therapy, sports therapy and rehabilitation; sports science	NA	NA	Analysis of the risk factors of disability-dependent injuries	NA	Volume and intensity of exercise, as well as the type of impairment, as the main risk factors of injury
Nowak et al., 2022 [31]; Comparative, survey-based study	International Journal of Environmental Research and Public Health; 4.614; Q2; Public health, environmental and occupational health	35; 27 males and 8 females; 14 ≤ 29 y; NA; Physical disability	NA	To evaluate the degree of adoption of healthy lifestyles	Self-report data	Experience, training volume and intensity, education, social, economic-financial, and employment status, and marital status were predictors of the uptake of health-related behaviors

**Table 3:** Main characteristics of the study included in the present scoping review concerning the biomechanics of para-rowing. Abbreviations: IF (Impact Factor); NA (not available); SCI (spinal cord injury); y (years).

Study, Study Design	Journal, IF (2022), Scimago Ranking, Subject Areas and Categories	Sample Size, Sex, Age, Experience, Type of Disability	Sports Class	Aim(s)	Outcome Measures	Findings
Severin et al., 2021 [6]; Case study	Frontiers in Sports and Active Living; NA; NA; NA	1; Female; 30 y; Elite; Physical disability (incomplete SCI)	PR1	To evaluate the effects of adjusting seat and backrest angle on performance, physiology, and biomechanics parameters	Rowing performance, physiology, and biomechanics	Outcomes improved
Schaffert and Mattes, 2015 [32]; Case series	Journal of Sports Sciences; 3.943; Q1; Orthopedics and sports medicine; physical therapy, sports therapy and rehabilitation; sports science	6; 3 males and 3 females; 34.8 ± 10.6 y; International level; Visual impairment	P3 mixed coxed four	To examine the effects of acoustic feedback on mean boat speed	Mean velocity, stroke rate, and power output	Outcomes improved
Held et al., 2022 [33]; Multiple single case in-field testing as a part of randomized crossover trial verified by a repeated measurement trial	Frontiers in Physiology; 4.755; Q1; Physiology and medical physiology	1; Male; 37 y; Elite; Physical disability	PR1	To examine the changing oar rotation axis position in indoor and in-field	Catch angle using a 3D motion capture system	Outcome increased
Cutler et al., 2017 [34]	Journal of Sports Sciences; 3.943; Q1; Orthopedics and sports medicine; physical therapy, sports therapy and rehabilitation; sports science	NA	NA	Comparison of kinematic movement patterns between able-bodied and adapted para-rowing set-ups	Handle pull force, kinematics stroke length, and posture (grip/landing angles on specific joints) plus data from literature	Greater trunk angle, higher stroke rate, and less vertical displacement in para rowers than able-bodied rowers

**Table 4:** Main characteristics of the study included in the present scoping review concerning emerging analytical approaches for kinematic assessments and predictions of mechanical outputs in para-rowing. Abbreviations: EMG (electromyography); IF (Impact Factor); NA (not available); y (years).

Study, Study Design	Journal, IF (2022), Scimago Ranking, Subject Areas and Categories	Sample Size, Sex, Age, Experience, Type of Disability	Sports Class	Aim(s)	Outcome Measures	Findings
Euiler and Finley, 2022 [35]; Modeling study	Journal of Sport Rehabilitation; 2.203; Q2–Q3; Orthopedics and sports medicine; physical therapy, sports therapy and rehabilitation; rehabilitation; sports science	10; 7 males and 3 females; 41.6 ± 13.4 y; various levels of experience; Physical disability	NA	To assess the reliability of upper-extremity muscle activity and kinematics during adaptive rowing	Muscle EMG assessment (peak muscle activity, mean muscle activity), and kinematics of the rowing stroke	Good to excellent reliability
Schwingel et al., 2009 [36]; Modeling study	Journal of Strength and Conditioning Research; 4.415; Q1; Medicine (miscellaneous); orthopedics and sports medicine; physical therapy, sports therapy and rehabilitation; sports science	9; Male; 30 ± 7.9 y; International level; Physical disability	NA	To assess the accuracy of predicting one repetition maximum (1RM) equations	Linear and exponential equation models (12 different equations)	Good accuracy for upper and lower body strength
Vieira et al., 2018 [37]; Modeling study	IEEE Transactions on Neural Systems and Rehabilitation Engineering; 4.528; Q1–Q2; Medicine (miscellaneous), internal medicine, neuroscience (miscellaneous), rehabilitation, biomedical engineering, computer science applications	11; NA; 23–47 y; International level; Physical disability	LTA-PD	Design and testing of a biomechanical model for the estimation of knee joint angle during indoor rowing	Knee angle changes (measured with inertial sensors)	High accuracy of the model (average error less than 2° compared to the traditional method)

**Table 5:** The ten most productive authors on para-rowing.

Author	Country	Number of Documents (%)	Number of Links	Total Link Strength	Author Cluster	Publication Year Range
Smoljanović T.	Croatia	3 (17.6%)	15	16	2	2011−2017
Porto Y.C.	Brazil	3 (17.6%)	10	14	1	2008−2014
Schwingel P.A.	Brazil	3 (17.6%)	10	14	1	2008−2014
Zoppi C.C.	Brazil	3 (17.6%)	10	14	1	2008−2014
Bojanić I.	Croatia	2 (11.8%)	8	9	2	2011−2013
Di Ciacca S.R.	Canada	1 (5.9%)	7	7	5	2017
Lebrun C.M.	Canada	1 (5.9%)	7	7	5	2017
Orlando K.	Canada	1 (5.9%)	7	7	5	2017
Thornton J.S.	Canada	1 (5.9%)	7	7	5	2017
Vinther A.	Denmark	1 (5.9%)	7	7	5	2017

**Table 6:** The main topological features of the scholarly community of authors on para-rowing.

Topological Feature	Value
Avg. Number of neighbors	6.75
Network diameter	2
Network radius	1
Characteristic path length	1.55
Clustering coefficient	0.94
Network density	0.45
Network heterogeneity	0.37
Network centralization	0.63
Connected components	13

## Data Availability

Not applicable.
